# Web-Based Textual Analysis of Free-Text Patient Experience Comments From a Survey in Primary Care

**DOI:** 10.2196/medinform.3783

**Published:** 2015-05-06

**Authors:** Inocencio Daniel Maramba, Antoinette Davey, Marc N Elliott, Martin Roberts, Martin Roland, Finlay Brown, Jenni Burt, Olga Boiko, John Campbell

**Affiliations:** ^1^Primary CareUniversity of Exeter Medical SchoolUniversity of ExeterExeterUnited Kingdom; ^2^RAND CorporationSanta Monica, CAUnited States; ^3^Collaboration for the Advancement of Medical Education Research and Assessment (CAMERA)Peninsula Schools of Medicine and DentistryPlymouth UniversityPlymouthUnited Kingdom; ^4^Institute of Public HealthUniversity of CambridgeCambridgeUnited Kingdom; ^5^Peninsula College of Medicine and DentistryUniversities of Plymouth and ExeterPlymouthUnited Kingdom

**Keywords:** patient experience, patient feedback, free-text comments, quantitative content analysis, textual analysis

## Abstract

**Background:**

Open-ended questions eliciting free-text comments have been widely adopted in surveys of patient experience. Analysis of free text comments can provide deeper or new insight, identify areas for action, and initiate further investigation. Also, they may be a promising way to progress from documentation of patient experience to achieving quality improvement. The usual methods of analyzing free-text comments are known to be time and resource intensive. To efficiently deal with a large amount of free-text, new methods of rapidly summarizing and characterizing the text are being explored.

**Objective:**

The aim of this study was to investigate the feasibility of using freely available Web-based text processing tools (text clouds, distinctive word extraction, key words in context) for extracting useful information from large amounts of free-text commentary about patient experience, as an alternative to more resource intensive analytic methods.

**Methods:**

We collected free-text responses to a broad, open-ended question on patients’ experience of primary care in a cross-sectional postal survey of patients recently consulting doctors in 25 English general practices. We encoded the responses to text files which were then uploaded to three Web-based textual processing tools. The tools we used were two text cloud creators: TagCrowd for unigrams, and Many Eyes for bigrams; and Voyant Tools, a Web-based reading tool that can extract distinctive words and perform Keyword in Context (KWIC) analysis. The association of patients’ experience scores with the occurrence of certain words was tested with logistic regression analysis. KWIC analysis was also performed to gain insight into the use of a significant word.

**Results:**

In total, 3426 free-text responses were received from 7721 patients (comment rate: 44.4%). The five most frequent words in the patients’ comments were “doctor”, “appointment”, “surgery”, “practice”, and “time”. The three most frequent two-word combinations were “reception staff”, “excellent service”, and “two weeks”. The regression analysis showed that the occurrence of the word “excellent” in the comments was significantly associated with a better patient experience (OR=1.96, 95%CI=1.63-2.34), while “rude” was significantly associated with a worse experience (OR=0.53, 95%CI=0.46-0.60). The KWIC results revealed that 49 of the 78 (63%) occurrences of the word “rude” in the comments were related to receptionists and 17(22%) were related to doctors.

**Conclusions:**

Web-based text processing tools can extract useful information from free-text comments and the output may serve as a springboard for further investigation. Text clouds, distinctive words extraction and KWIC analysis show promise in quick evaluation of unstructured patient feedback. The results are easily understandable, but may require further probing such as KWIC analysis to establish the context. Future research should explore whether more sophisticated methods of textual analysis (eg, sentiment analysis, natural language processing) could add additional levels of understanding.

## Introduction

Patient experience is an important component of quality of health care, and questionnaires capturing patient experience have been widely used to provide insight into the quality of primary health care provision [1-3]. Feedback from survey results has been proposed as a cost-effective method to support and facilitate quality improvement [4,5].

In addition to capturing responses via closed questionnaire items, open-ended questions eliciting free-text comments have also been widely adopted [6,7] as the exclusive use of quantitative data limits the potential of surveys to improve practice [8]. Analysis of free-text comments can provide deeper or new insight, identify areas for action, and initiate further investigation [9]. Also, they may be a promising way to progress from documentation of patient experience to achieving quality improvement [9,10]. Free-text comments have been evaluated using methods such as content analysis [11,12], thematic analysis [9,13,14], and the Holsti Method [15]. However, these approaches can be resource intensive [6,15,16]. To efficiently deal with a large amount of free-text, new methods of rapidly summarizing and characterizing the text are being explored [17].

Text clouds are visual representation of a body of text, where the more frequently occurring words appear larger in the "cloud"[18,19]. The first widespread use of text clouds was “tag clouds”, which originated as a representation of the "tags" or keywords that users would assign to a Web resource [20,21]. Tag clouds have been used in health related websites to counter biased information processing [22].

The same technology that creates tag clouds may also be used to create word clouds from texts and textual data in general [23]. Text clouds differ from tag clouds in that their purpose is predominantly comprehension of the text rather than navigation of webpages [23]. Text clouds can be used to rapidly summarize textual data, revealing textual messages in a pictorial form [24]. Text clouds may have utility in supporting searching and browsing of webpages, as well as impression formation and recognition/matching of textual data [25].

Web applications such as TagCrowd, Many Eyes, Wordle, and Tagxedo are commonly used to generate text clouds. The majority of these are free for nonprofit use.

Text clouds have been used in a wide range of health related areas, such as examining the differences between various versions of a General Medical Council document [24] as well as a UK Government White Paper [26], survey responses on ehealth [27], survey of pharmacists’ perceptions [28], patients’ use of online message forums [29], and to analyze the responses of multiple sclerosis sufferers to open-ended questions [30].

Other uses of computerized textual analysis in health include: automatic analysis of online discussions related to diabetes [31], content analysis of the free text comments in multi-source feedback about specialist registrars [32], automatic drug side effect discovery by analysis of online patient submitted reviews [33], keyword analysis of an online survey investigating nurses' perceptions of spirituality and spiritual care [34], and uncovering signs and symptoms of opiate exposure from comments posted on YouTube [35].

We aimed to investigate the feasibility of using Web-based textual analysis for extracting useful information from large amounts of free-text patient comments, and to identify key issues or topics that would be revealed by computerized text processing, using tools that are currently available at no cost on the Web.

##  Methods

### Participants and Procedure

The data was collected as part of the “Improve” study, a research program funded by the National Institute for Health Research (NIHR) [36], exploring various aspects of patient experience in primary care. One of the projects involved a post-consultation postal survey using a modified version of the English GP Patient Survey (GPPS) questionnaire. The GPPS is the largest survey program of patients registered with an English general practice. A random sample of patients from each English practice-~2.6 million patients each year in total-is invited to take part in the survey [37,38]. A particular change made to the GPPS questionnaire (at the request of participating practices) was the inclusion of a free-text comments question worded as follows: "*Your [general] practice has asked that we collect any further comments you would like to make about the service they provide*."

Detailed survey methods have been previously reported in the paper by Roberts et al [39], which are briefly summarized here. Following a recent face-to-face consultation between November 2011 and June 2013 with one of 105 doctors from 25 practices in six areas of England (Cornwall, Devon, Bristol, Bedfordshire, Cambridgeshire, Peterborough, and North London), patients were sent a questionnaire regarding their experiences of care. One reminder was sent to nonrespondents. Free-text comments were anonymized during data entry, extracted from the database and exported to a text file. Approval for the study was obtained from the South West 2 Research Ethics Committee on January 28^th^ 2011 (ref: 09/H0202/65). Return of a completed questionnaire was taken to indicate patient consent to participate in the study.

### Textual Analysis Methods

Free-text comments were analyzed using three Web-based textual analysis tools: TagCrowd v.10/02/2011 [40], Many Eyes v.1.0 [41], and Voyant Tools v.1.0 [42], which were chosen for their ease of use and range of functionalities.

TagCrowd is a Web application for visualizing word frequencies in any text by creating what is popularly known as a word cloud, text cloud or tag cloud. We created text clouds based on an aggregated corpus of free-text patient comments.

We used the following parameters in TagCrowd: (1) frequently occurring English words and connectives (eg, “a”, “in”, “is”, “it”, and “you”) were ignored; (2) the tag cloud was created from the 50 most frequently occurring single words; (3) a stemming algorithm combined related words (eg, learn, learned, learning -> learn). The 50 word limit was chosen as it has been used in previous work using text clouds to examine health information [24,26]. We also tried generating a 60 word text cloud but found the result to be difficult to read.

Many Eyes is a Web-based data visualization application created by IBM [41]. Fundamentally the software incorporates the capacity to create and view various forms of text visualization and representation. We chose to use Many Eyes because of its capability of creating text clouds from the most frequent two-word combinations. We hypothesized that two-word combinations might give a more nuanced insight into the meanings behind the most frequently used words as some of their associations would be preserved.

Voyant Tools is a Web-based reading and analysis environment for digital texts. It was created as part of a collaborative project to develop and theorize text analysis tools and text analysis rhetoric [42]. In addition to calculating word frequencies and creating text clouds, Voyant Tools performs other textual analysis functions, such as identifying distinctive words in the documents that make up a text corpus. To investigate the validity of the distinctive words component, we divided the comments into separate text files depending on whether the patients reported if they were either “satisfied” or “not satisfied” with their experience of care. The question was “In general, how satisfied are you with the care you get at this GP surgery or health center?”. Patients were given five options to rate their satisfaction with the practice: “very satisfied”, “fairly satisfied”, “neither satisfied nor dissatisfied”, “fairly dissatisfied”, and “very dissatisfied”. In this analysis, the “very satisfied” and “fairly satisfied” responses were recoded as “satisfied” and the last three options as “not satisfied”. We used the “distinctive words” function to identify the words that occurred more frequently in comments originating from patients who were “satisfied” and words which occurred more frequently in comments from patients who were “not satisfied”.

### Statistical Analysis

We used logistic regression to investigate the occurrence/nonoccurrence of words within individual patient comments. The words were selected from the results of the distinctive word analysis. We obtained and compared the frequency of use of the five most distinctive words from the comments classified as originating from either the “satisfied” or “not satisfied” patients (ten words in total).

Logistic regression was used to predict the presence or absence of each of these words in a comment from the standardized scores (z-scores) of the patients’ responses to the survey question on satisfaction. We used the following formula for the standardized scores (z-scores):

z=(x-μ)/(σ)

Two additional models were run for each word, predicting its presence or absence from the z-scores of the patients’ ratings of their confidence and trust in the doctor and their ratings of the doctors’ communication skills. We derived these scores from the patients’ responses to two other structured questions that were asked in the questionnaire. These variables were chosen as we hypothesized that confidence and trust in the doctor, as well as the communication skills of the doctor could influence the words used by the patients in their comments. Statistical analyses were performed in STATA version SE13.1 for Windows. We then plotted the odds ratios for the selected words against their standardized frequencies. The standardized frequency is calculated in the same way as a z-score, where x is the frequency of a particular word, *μ* is the mean frequency of all words in the patient comments, and *σ * is their standard deviation.

### Keyword in Context Analysis

Voyant Tools provides a Keyword in Context (KWIC) function. KWIC involves searching for a particular keyword in the text and analyzing its local meaning in relation to a fixed number of words immediately preceding and following it [43]. KWIC can help identify underlying connections that are being implied by the text [44]. KWIC analysis had been used in content analysis of blogs about female incontinence [45], as well as in content analysis of audiology service improvement documentation [46]. The KWIC function in Voyant tools can quickly display the KWIC for a selected keyword and the results can be exported to a format suitable for further analysis. For this analysis we selected 15 words that preceded and followed the word “rude”. The resulting text was then manually examined to determine the context of the use of “rude”, and the results were tabulated.

##  Results

### Textual Analysis Methods

**Figure 1:**
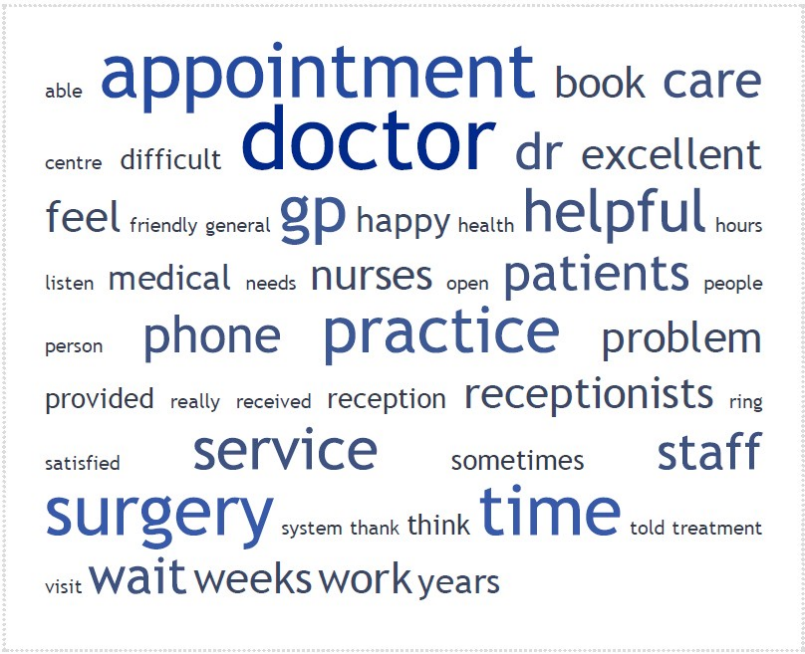
Single-word text cloud created in TagCrowd from all free text comments.

From 7721 respondents, we collected 3426 individual comments (comment rate: 44.4%). The comments came to a total of 150,699 words of which 6867 are unique words. The average length of response is 43.98 words. There are 273 instances of 90 unique, non-English terms (mostly misspellings). Figure 1 shows the text cloud resulting from all the free-text comments as generated by TagCrowd. The five most frequent words were: “doctor”, “appointment”, “surgery”, “practice”, and “time”. Included in the 50 most frequent words were those that have a positive connotation such as: “helpful” and “excellent”. Words with a negative connotation, such as “difficult” and “problem” were also present, but were less frequent.

**Figure 2:**
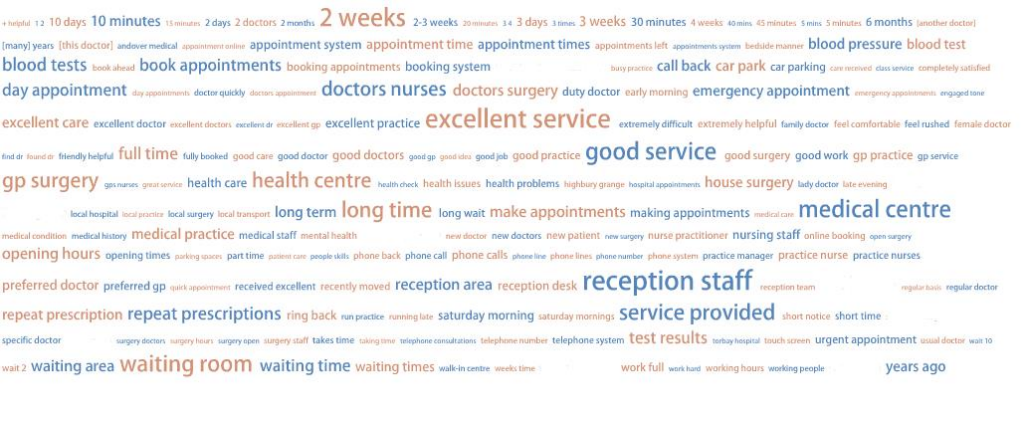
Two-word text cloud created in Many Eyes.

The two-word text cloud generated by Many Eyes is shown in Figure 2, displaying the 200 most frequent two-word phrases (bigrams). The five most frequent bigrams were: “reception staff”, “excellent service”, “two weeks”, “medical centre” and “good service”.

**Figure 3:**
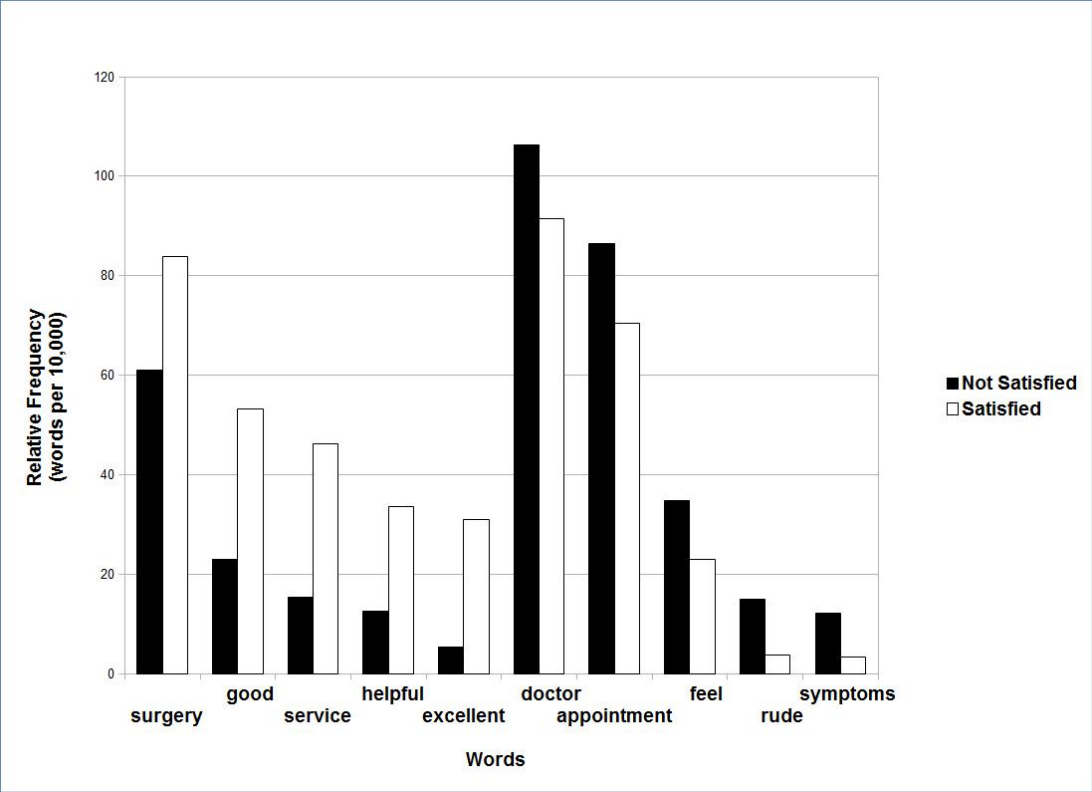
Word frequency by patient satisfaction.

Figure 3 shows the results of the Voyant Tools distinctive words component when applied to comments categorized as originating from satisfied or dissatisfied patients. The words “surgery”, ”excellent”, “service”, “good”, and “helpful” were the five most distinctive words from satisfied patients, while the words “doctor”, “feel “, “appointment”, “rude”, and “symptoms” were the five most distinctive words in the comments from dissatisfied patients.

### Statistical Analysis

From the logistic regression models, odds ratios were calculated for the distinctive words. In this analysis, the odds ratio indicates the amount by which the odds of a particular word occurring at least once in a comment are multiplied for every point increase in the z-score. Table 1 reports the results of the logistic regression for the 10 distinctive words and the scores for satisfaction, doctor-patient communication, and confidence and trust of the patient in the doctor.

**Table 1:** Odds ratio (95% CI, R^2^ value) for the occurrence of distinctive words corresponding to a one standard deviation increase in measures of patient experience.

Word	Overall satisfaction N=3134 OR (CI, R^2^)	Doctor’s communication skills N=3062 OR (CI, R^2^)	Confidence and trust in the doctor N=3066 OR (CI, R^2^)
Service	1.39 (1.25-1.54, .02)	1.25 (1.13-1.39, .007)	1.29 (1.16-1.43, .009)
Good	1.11 (1.02-1.21, .002)	1.09 (0.99-1.2, .001)	1.06 (0.97-1.16, .0005)
Excellent	1.96 (1.63-2.34, .04)	2.09 (1.69-2.58, .09)	1.76 (1.45-2.15, .003)
Surgery	0.94 (0.88-1.01, .0008)	0.98 (0.90-1.05, .0001)	1.00 (0.93-1.08, .00)
Helpful	1.19 (1.07-1.32, .005)	1.24 (1.10-1.40, .006)	1.10 (0.99-1.22, .001)
Appointment	0.67 (0.63-0.72, .04)	0.77 (0.77-0.82, .01)	0.80 (0.74-0.86, .01)
Doctor	0.76 (0.71-0.81, .02)	0.81 (0.75-0.87, .01)	0. 81 (0.76-0.88, .008)
Feel	0.79 (0. 72-0.87, .01)	0.78 (0.71-0.86, .01)	0.77 (0.70-0.85, .01)
Rude	0.53 (0.46-0.60, .10)	0.63 (0.55-0.74, .04)	0.60 (0.51-0.70, .05)
Symptoms	0.60 (0.51-0.70, .06)	0.61 (0.52-0.72, .05)	0.64 (0.54-0.77, .03)

As shown in the table, the regression for the word “excellent” results in an OR of 1.96, for the bivariate model for patient satisfaction; that is, an increase of one standard deviation in the patient satisfaction is associated with almost twice the odds of the word “excellent” occurring in the comments. There is also a significant association of the occurrence of “excellent” in the comments with the z-scores for doctor communication skills and confidence in the doctor which have odds ratios of 2.09 and 1.76 respectively.

In contrast, the word “rude” has an OR of 0.53 in the bivariate model for patient satisfaction, indicating that an increase in one standard deviation in the satisfaction score almost halves the odds that the word “rude” will appear in the comments. The OR is also significantly lower for the occurrence of “rude” when scores for doctor communication skills or confidence in the doctor are higher. To summarize, the words “service” and “excellent” had a significant positive association for overall satisfaction, doctor's communication skills scores, and confidence and trust in the doctor scores. The word “helpful” had a significant positive association for overall satisfaction and doctor’s communication skill scores, but was not significant for confidence and trust. The words “rude” and “symptoms” had a significant negative association with all three scores.

**Figure 4:**
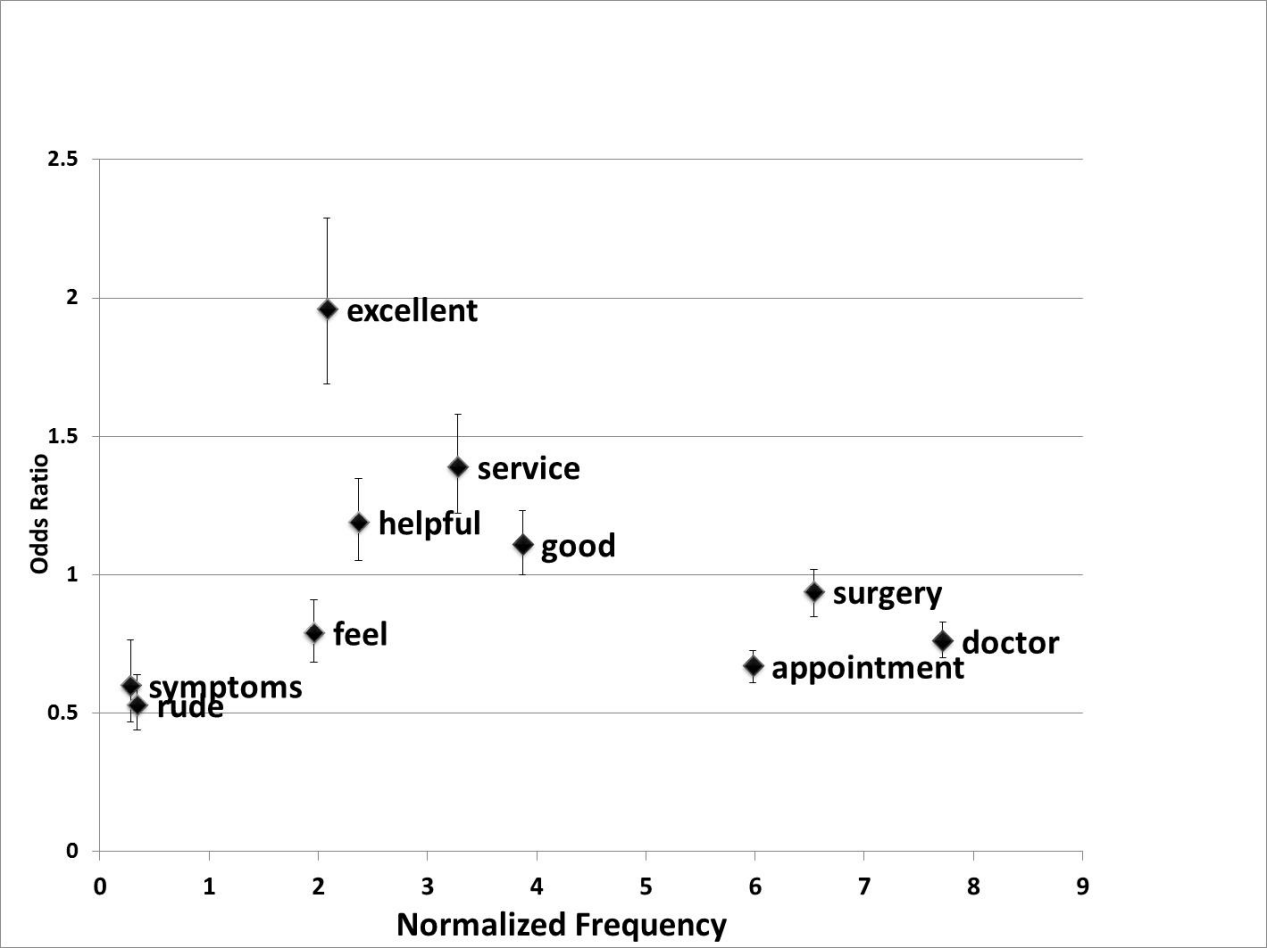
Frequency of selected words and the odds ratios (with 95% confidence intervals) associated with the z-scores for satisfaction.

Figure 4 shows a plot of the odds ratios for the occurrence of a word due to a one standard deviation increase in patient satisfaction score as calculated by the bivariate model. The odds ratios for ten distinctive words (five most distinctive words each from satisfied and dissatisfied patients) are plotted against their standardized frequencies (x-axis).

###  Keywords in Context (KWIC) Analysis

We chose to look at the context of the usage of the word “rude” using Keyword in Context (KWIC) analysis. We examined 15 words to the left and right of the keyword in question because we felt that was the minimum amount where we could satisfactorily establish the context of use of the word in question. Earlier attempts using 10 words on each side gave results wherein the context was still ambiguous in some of the comments. We manually reviewed the output from the KWIC tool, and established the context of the various instances of the adjective “rude”. We then constructed a table listing the sources of the rude behavior and their frequency of mention (Table 2).

Overall, “reception staff” was mentioned in 63% of the occurrences of the word “rude”, while doctors accounted for 22% of occurrences. Among the patients who were dissatisfied, the proportion of doctors being associated with occurrences of the word “rude” increased to 30% compared to 15% in satisfied patients. Reception staff had a larger proportion of association with occurrences of the word “rude” among satisfied patients, 72%, than in patients who were dissatisfied: 54%.

**Table 2:** Keyword in Context (KWIC) analysis for the word “rude”. Frequency of occurrence (% within patient type) by patient satisfaction and subject.

	Subject of adjective “rude”						
	Frequency of occurrence (% within patient type)						
Patients	Doctor	Nurse	Practice manager	Reception staff	Staff	Patient	Total
Not satisfied	11 (30)	1 (3)	2 (5)	20 (54)	3 (8)	0 (0)	37
Satisfied	6 (15)	3 (7)	1 (2)	29 (72)	1 (2)	1 (2)	41
All	17 (22)	4 (5)	3 (4)	49 (63)	4 (5)	1( 1)	78

## Discussion

### Principal Results

We found the three textual analysis tools easy to use and the results were generated very quickly, considering the volume of text that was processed (approximately 150,699 words). The tools used the standard ASCII text file format, which most data analysis software can easily export to. The text clouds did give a concise summary of what the majority of comments were about, but it was difficult to establish the exact context of the use of the most frequent words. The distinctive word analysis gave more insight by showing the different usage of words by differing sources of comments (satisfied vs dissatisfied patients). Some of the words in the output of the distinctive word analysis showed significant associations with satisfaction scores, notably, “excellent” and “rude”. The word “excellent” was associated with high patient satisfaction scores. The high frequency of the two-word phrase “excellent service” showed that patients used it in relation to their rating of the quality of service they received.

The word “rude” occurred much more frequently in comments from dissatisfied patients, suggesting that rude behavior encountered by patients may trigger dissatisfaction. The KWIC analysis showed that the word “rude”, was most commonly associated with the reception staff. Receptionists have been recognized as crucial members of the primary health care team [47], and recent work has suggested that the historical perception of the receptionist as a “dragon behind the desk” has been getting in the way of understanding the role of receptionists and thus improving patient care [48]. Also worth noting is that the proportion of rude actions being attributed to doctors was higher amongst the patients who were dissatisfied with their practice. Winsted has identified rudeness from doctors and other forms of negative behavior as being a “dissatisfier” in medical encounters [49].

The increased relative frequency of the word “feel” in the comments from dissatisfied patients might indicate an emotional reaction being a component of patient dissatisfaction. However, the word may also be used in other contexts, for instance “I feel that the doctor should”, as opposed to “I feel disappointed.” The recurrence of the word “symptoms” in the comments from dissatisfied patients could indicate a relationship between dissatisfaction and the perception of poor health, as has been reported previously by Xiao et al [50]. It may also provide a comment on the perceived thoroughness of the clinical encounter. One point of interest is that the words positively associated with patient satisfaction focus on the system (eg, “excellent service”), while those associated with dissatisfaction highlight some of the interpersonal aspects of care (eg, “rude”, “feel”).

### Limitations

While the textual analysis applications are easy to use and give results quickly, one limitation is that an internet connection is required for all the software tools to work. However, a high-speed connection is not necessary, and the software runs on any modern operating system with an updated Web browser.

When we attempted to identify the messages contained in the text cloud, we found it difficult to ascertain the significance of the high frequency of the words “doctor”, “practice”, “surgery”, “appointment”, and “time”. This is due to the text cloud showing the words dissociated from their original context, making it difficult to discern the meaning behind the high frequencies of these words. This loss of context due to the dissociation of the words from one another is a major limitation in the interpretation of the results of the text cloud. When words are separated from one another, and only their frequencies rather than their relationships are scrutinized, there is a danger of overlooking subtle and important nuances and meanings formed by the synergy of the words [24]. The software tools are also limited in that they are unable to group together words that are synonymous, (eg, “doctor”, “dr”, and “gp”), unless the software is specifically instructed to group these synonyms. In addition to the individual words, meaning is also conveyed by the patterns that words form.

Another consequence of dissociation is that our method does not automatically deal with negation of terms. However, for the words “good”, “excellent”, “helpful” “rude”, and “symptoms”, we examined the results of the keyword in context extraction to see if they contained instances of negation. We were satisfied that all mentions of those words did not contain instances of negation. A more sophisticated approach using natural language processing and machine learning is required to automatically deal with negation. Sentiment analysis, which is a more sophisticated textual analysis technique, is one method that takes the patterns of words, and not just their frequencies, into account. Research reports are emerging in which sentiment analysis has been used to examine free text comments from patients [51-57].

A further limitation of this study is related to the nature of the question being presented to elicit the comments. The very broad nature of the request for comments means that the patients’ responses were, almost inevitably, quite varied. The wide spectrum of issues raised in the comments make them quite difficult to neatly categorize and characterize. The quality of information gleaned from patient responses could be improved by focusing the wording of the request for comments to address central issues of interest [9]. This focus of interest could also be coupled to particular quantitative questions, to give insight into why the patient answered the question in that particular way.

### Further Research

Web-based textual analysis shows promise as a means of rapidly summarizing the messages contained in free-text comments from primary care patients. Text clouds are a feasible means of presenting the most frequent words used in free-text comments from patients. However, text clouds are limited by an inability to provide a contextually meaningful summary of the original corpus of comments. This is commonly encountered when relying primarily on a simple, mechanistic algorithm, in this case, word frequency. Words convey meaning by working together, and there is a synergy created through the combination of various words [24]. A more accurate way of capturing the messages contained in the free-text comments by a computer mediated approach is through KWIC analysis. The use of more sophisticated technologies, such as machine learning, natural language processing, neural networks, and sentiment analysis may address some of these shortcomings. Future research needs to be done around generating sentence level summarization using the techniques from the NLP community [58], such as latent Dirichlet allocation [59,60] . For a wider uptake, a user-friendly (preferably open source) application needs to be developed to fill this gap. This would enable practices to make better use of the large amounts of free-text feedback that they have collected. In addition, careful attention needs to be paid to formulating focused and precise requests for comments which might be expected to yield feedback that could provide a substantial basis for computer mediated textual analysis. Finally, mixed methods approaches as well as sociocybernetics methods have also been proposed as a way of completing the picture of patient experience [61,62].

### Conclusions

Our study has shown that by using Web-based text processing tools to extract information from patient comments, we can discover words that the patients have used in their comments that have significant associations with quantitative measurements of patient experience. The logistic regression revealed strong positive and negative associations between the satisfaction scores and the occurrence of certain words. KWIC analysis was then used to examine the context of the uses of words, which yielded useful information; for example, the sources of rude behavior that is associated with patient dissatisfaction. This approach could help practices in formulate policies to increase patient satisfaction. Sequential use of these methods may prove useful in documenting how patients’ experience of care changes over time, similar to the method used by Gill et al in revealing the longitudinal changes in the document “Good Medical Practice” produced by the General Medical Council [24]. An approach that examines the key words in the context is useful in deriving insights from the free-text comments. Further research is necessary in refining these methods, so that the results would be comparable to traditional techniques of content analysis.

## Multimedia Appendix 1

Screenshots of Web based textual analysis tools.

## Multimedia Appendix 2

Survey questionnaire.

## Abbreviations

ASCII: American Standard Code for Information Interchange
CI: confidence interval
GPPS: General Practice Patient Survey
KWIC: keyword in context
NHS England: National Health Service England
NIHR: National Institute for Health Research
NLP: natural language processing
OR: odds ratio
