# Supplementary material for: Web-Based Textual Analysis of Free-Text Patient Experience Comments From a Survey in Primary Care
Source: JMIR Med Inform. 2015 May 6;3(2):e20. doi: 10.2196/medinform.3783 (PMC4439523; doi:10.2196/medinform.3783)
Supplement: Supplementary file 1 [file medinform_v3i2e20_app1.pdf]

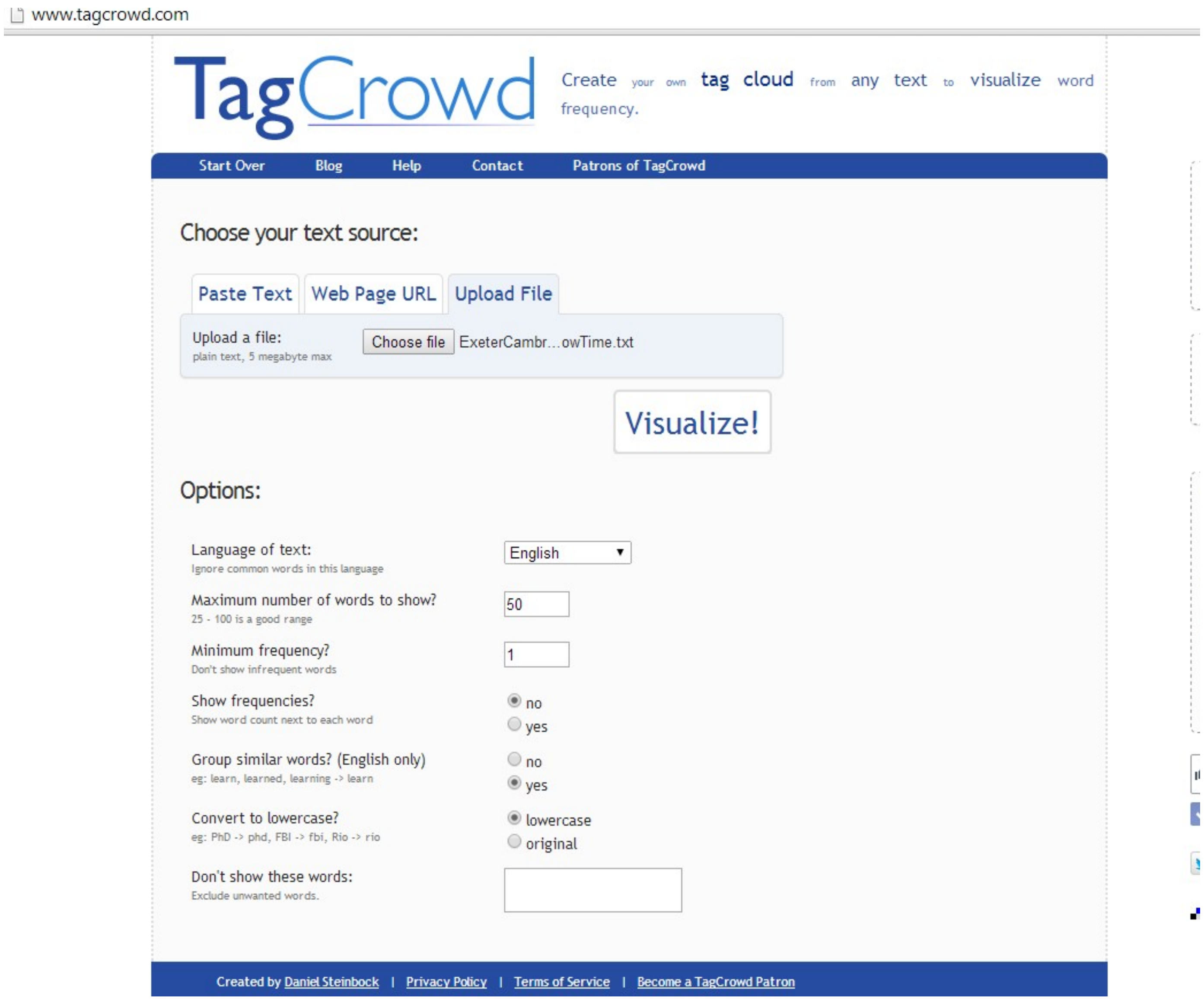

Tag Crowd: 1) Select settings

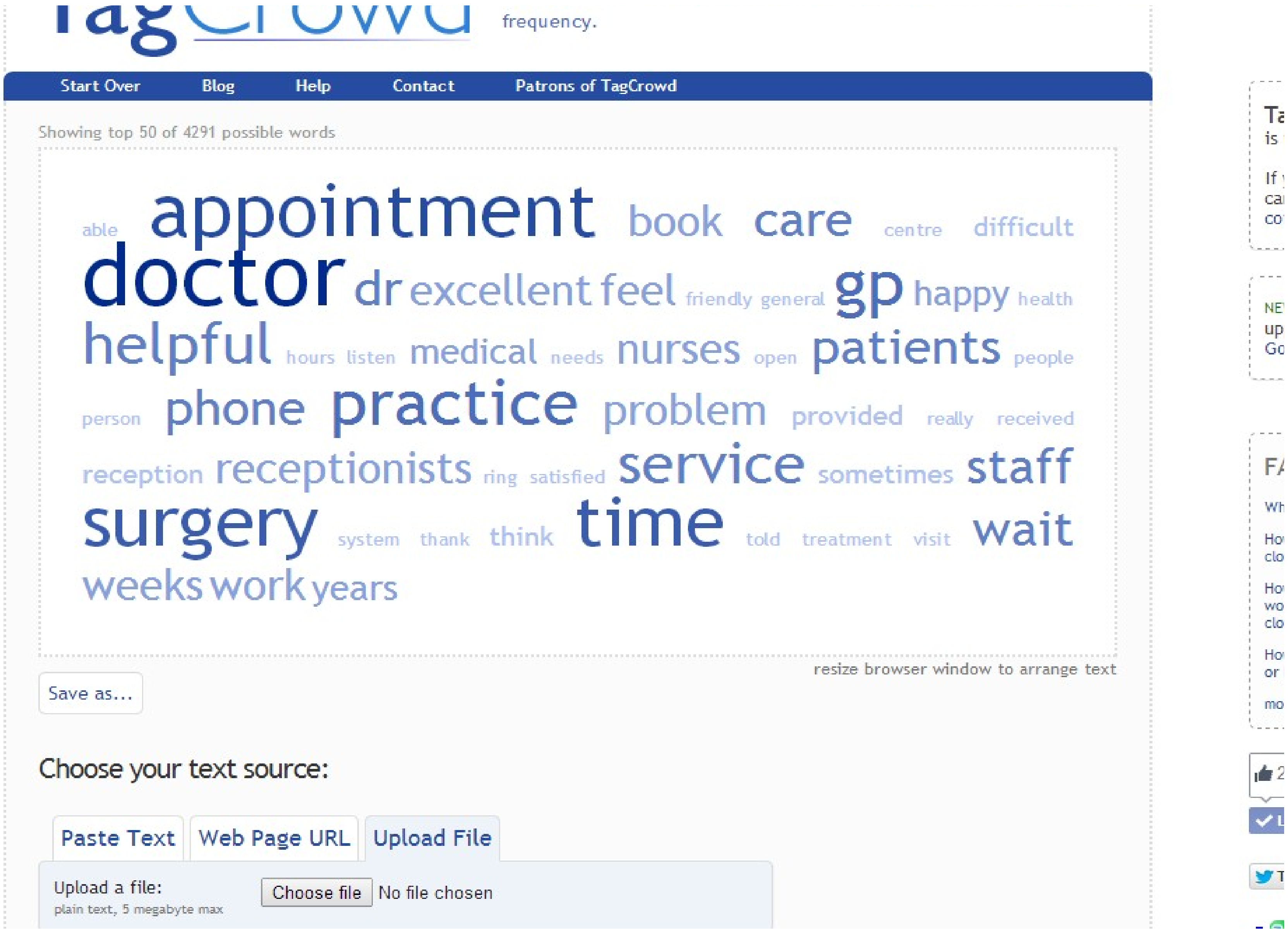

Tag Crowd: 2) Generate text cloud

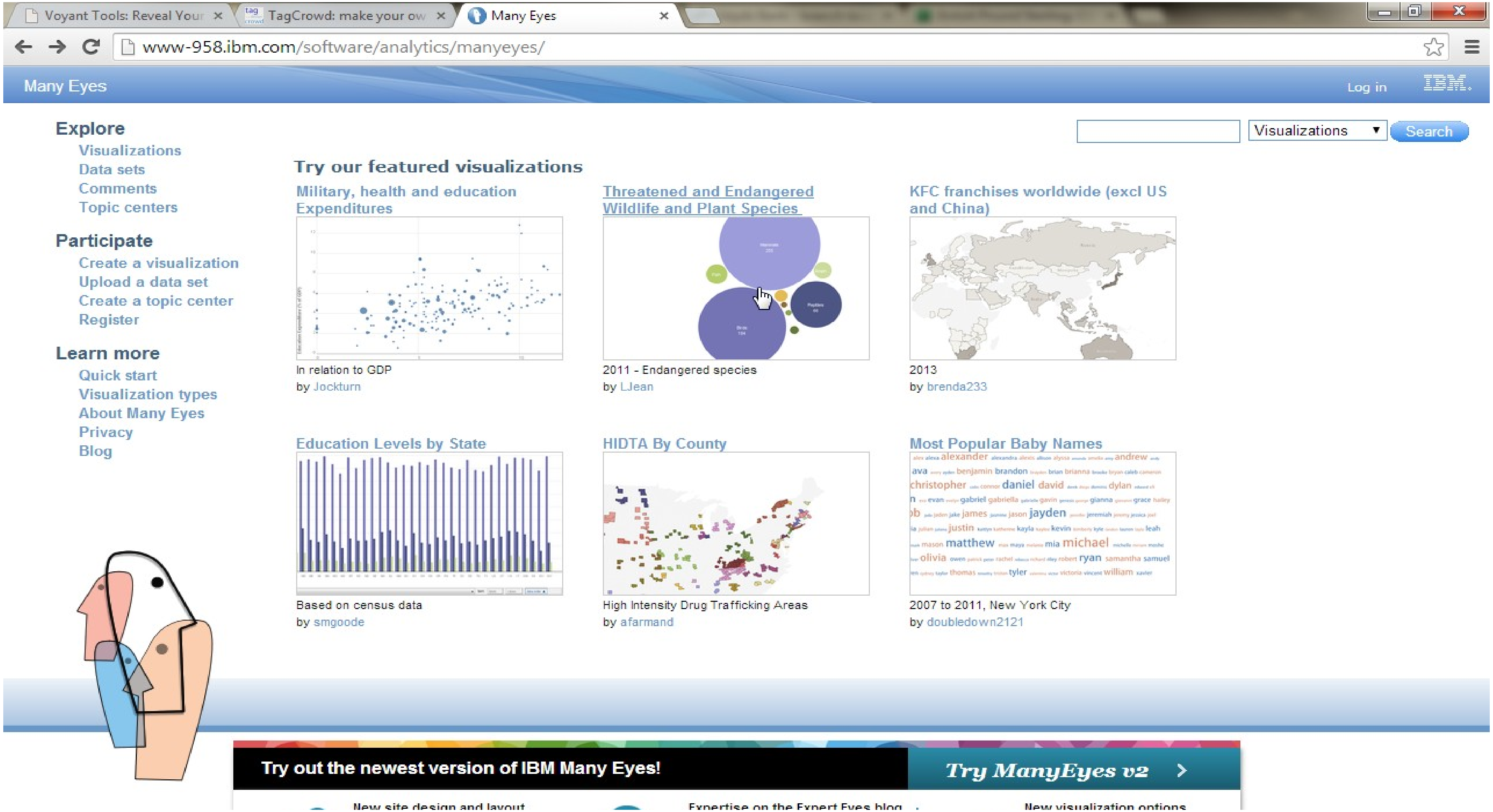

Many Eyes welcome page

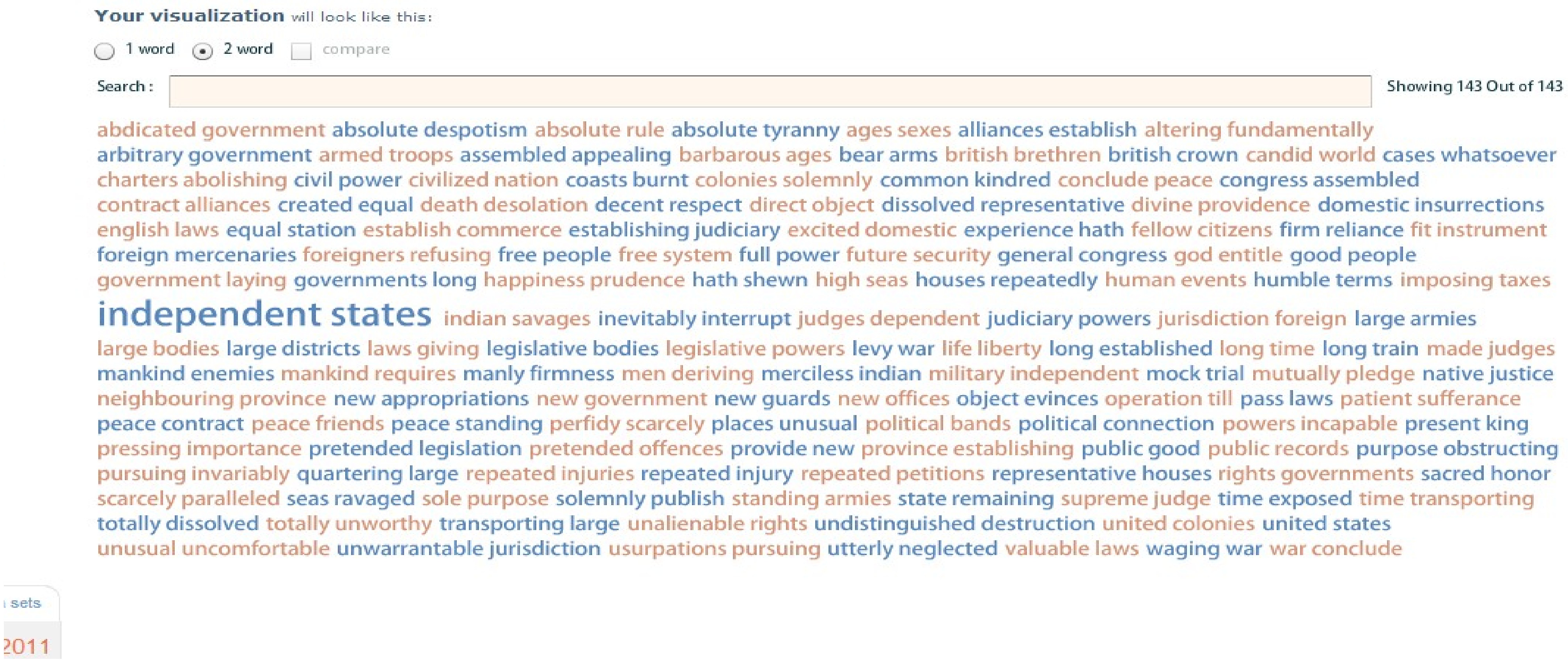

Example of two-word text cloud

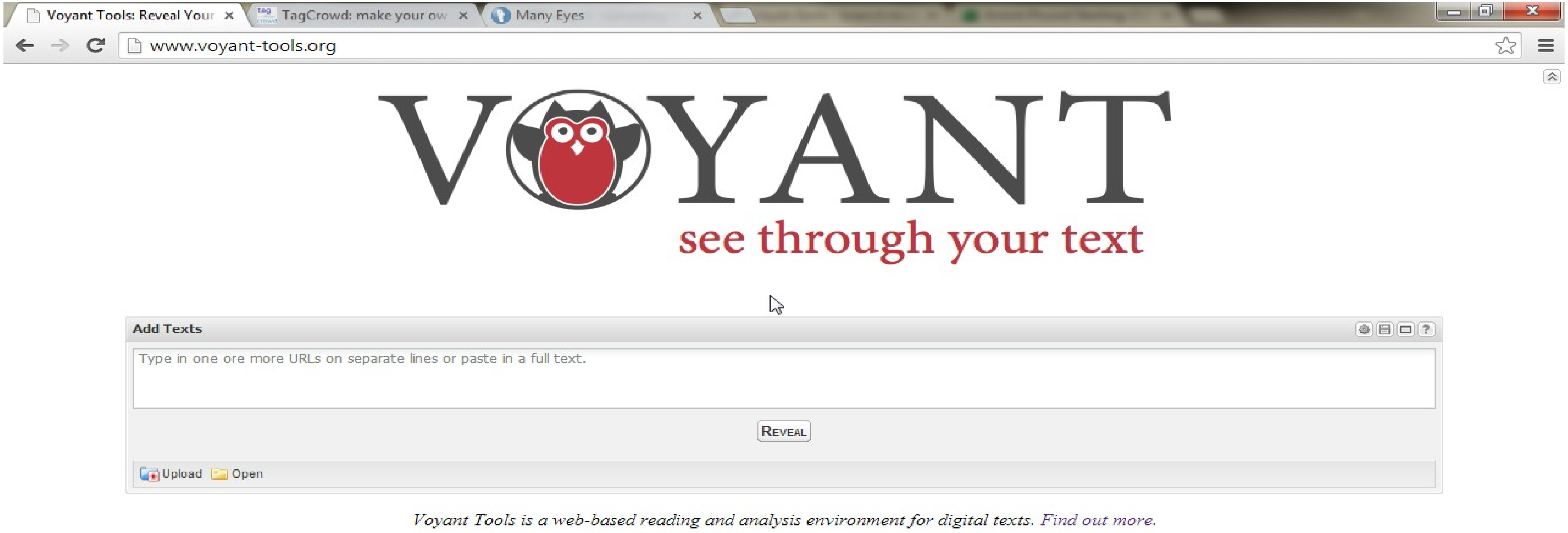

Opening page of Voyant tools

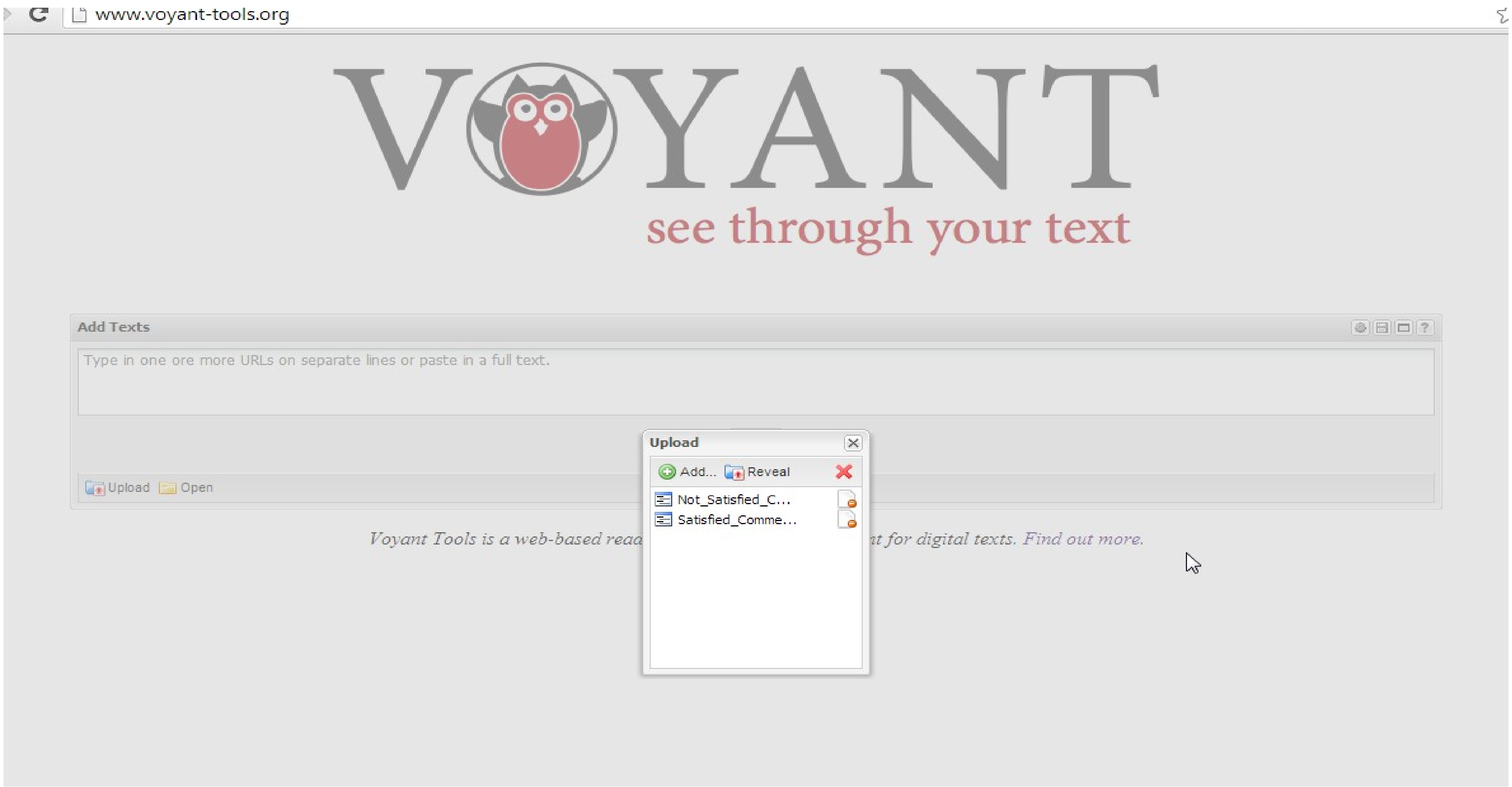

Uploading text files to Voyant tools

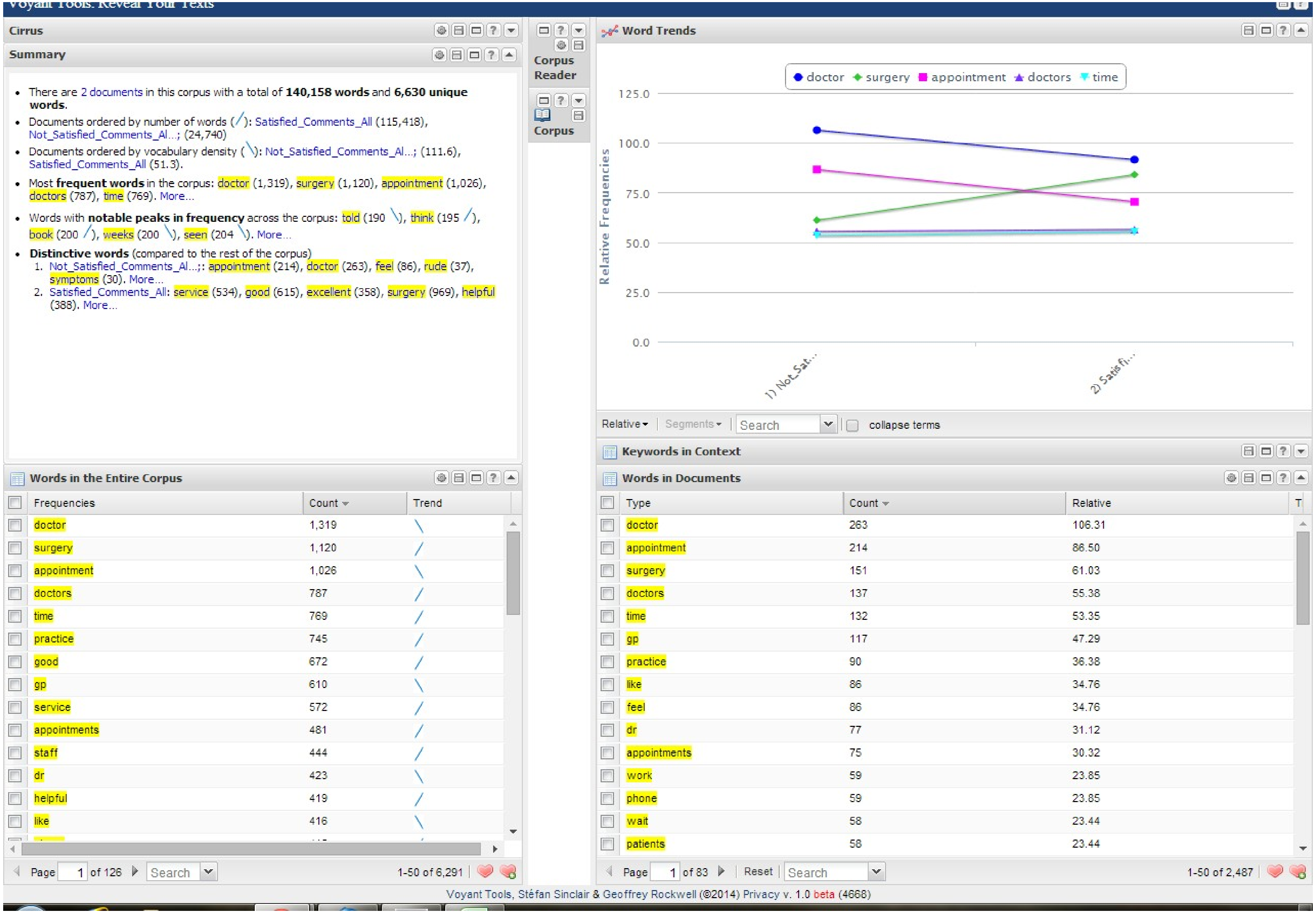

Voyant tools overview

Voyant Tools: Reveal Your Texts

Summary

- There are 2 documents in this corpus with a total of **140,158 words** and **6,630 unique words**.
- Documents ordered by number of words (/): Satisfied_Comments_All (115,418), Not_Satisfied_Comments_Al...; (24,740)
- Documents ordered by vocabulary density (\): Not_Satisfied_Comments_Al...; (111.6), Satisfied_Comments_All (51.3).
- Most **frequent words** in the corpus: doctor (1,319), surgery (1,120), appointment (1,026), doctors (787), time (769). More...
- Words with **notable peaks in frequency** across the corpus: told (190 \), think (195 /), book (200 /), weeks (200 \), seen (204 \). More...
- **Distinctive words** (compared to the rest of the corpus)
  1. Not_Satisfied_Comments_Al...;: appointment (214), doctor (263), feel (86), rude (37), symptoms (30). More...
  2. Satisfied_Comments_All: service (534), good (615), excellent (358), surgery (969), helpful (388). More...

Voyant tools: Distinctive words

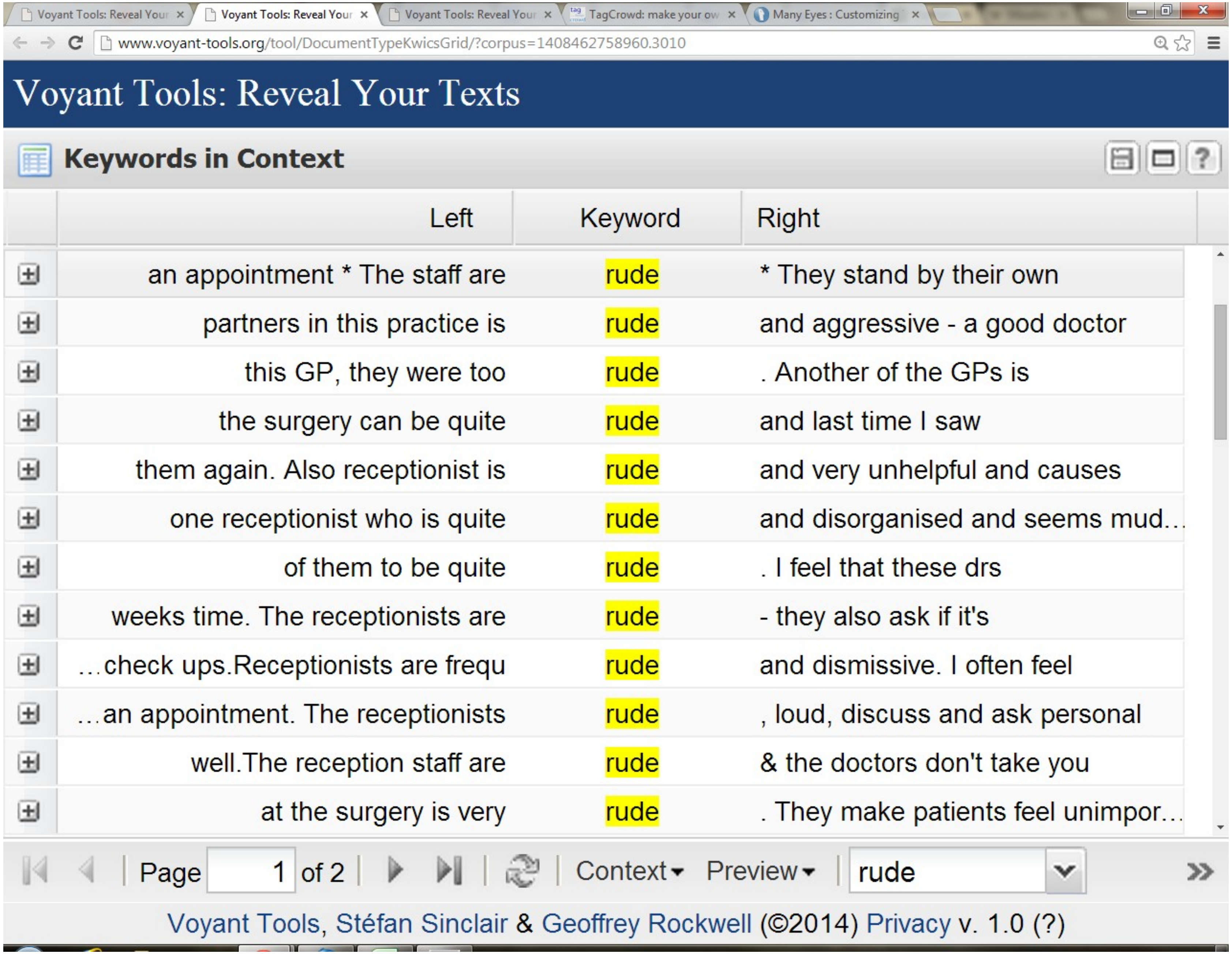

Voyant tools: Keywords in context (KWIC)
