# Supplementary material for: Web-Based Textual Analysis of Free-Text Patient Experience Comments From a Survey in Primary Care
Source: JMIR Med Inform. 2015 May 6;3(2):e20. doi: 10.2196/medinform.3783 (PMC4439523; doi:10.2196/medinform.3783)
Supplement: Supplementary file 2 [file medinform_v3i2e20_app2.pdf]

PENINSULA COLLEGE OF MEDICINE & DENTISTRY
UNIVERSITIES OF EXETER & PLYMOUTH

improve

UNIVERSITY OF CAMBRIDGE

SEEING THE DOCTOR

Please answer the questions below by putting an ✕ in ONE BOX for each question unless more than one answer is allowed (these questions are clearly marked). We will keep your answers completely confidential.

A. APPOINTMENTS AT YOUR GP SURGERY OR HEALTH CENTRE

Q1 How do you normally book your appointments to see a doctor or nurse at this GP surgery or health centre?

Please ✕ all the boxes that apply to you

- ☐ In person
- ☐ By phone
- ☐ By fax machine
- ☐ Online
- ☐ Digital TV
- ☐ Doesn't apply

Q2 Which of the following methods would you prefer to use to book appointments at this GP surgery or health centre?

Please ✕ all the boxes that apply to you

- ☐ In person
- ☐ By phone
- ☐ By fax machine
- ☐ Online
- ☐ Digital TV
- ☐ No preference

B. GETTING THROUGH ON THE PHONE

Now please think about times you have phoned this GP surgery or health centre in the past 6 months.

Q3 In the past 6 months, how easy have you found the following?

Please put an ✕ in one box for each row

	Haven't tried	Very easy	Fairly easy	Not very easy	Not at all easy	Don't know
Getting through on the phone	☐	☐	☐	☐	☐	☐
Speaking to a doctor on the phone	☐	☐	☐	☐	☐	☐
Speaking to a nurse on the phone	☐	☐	☐	☐	☐	☐
Getting test results on the phone	☐	☐	☐	☐	☐	☐

C. SEEING A DOCTOR

Q4 In the past 6 months, have you tried to see a doctor fairly quickly?
By 'fairly quickly' we mean on the same day or in the next 2 weekdays the GP surgery or health centre was open.

- ☐ Yes....................... Please go to Q5
- ☐ No........................ Please go to Q7
- ☐ Can't remember.... Please go to Q7

Q5 **Think about the last time you tried to see a doctor fairly quickly. <u>Were you able to</u> see a doctor on the same day or in the next 2 weekdays the GP surgery or health centre was open?**

- ☐ Yes........................ Please go to Q7
- ☐ No .... Please go to Q6
- ☐ Can't remember.... Please go to Q7

Q6 **If you couldn't be seen within the next 2 weekdays the GP surgery or health centre was open, why was that?**

Please ✕ <u>all</u> the boxes that apply to you

- ☐ There weren't any appointments
- ☐ The times offered didn't suit me
- ☐ The appointment was with a doctor I didn't want to see
- ☐ I could have seen a nurse but I wanted to see a doctor
- ☐ I was offered an appointment at a different branch of my surgery
- ☐ Another reason
- ☐ Can't remember

Q7 **In the past 6 months, have you <u>tried</u> to book ahead for an appointment with a doctor?**
By 'booking ahead' we mean booking an appointment more than 2 full weekdays in advance.

- ☐ Yes........................ Please go to Q8
- ☐ No .... Please go to Q9
- ☐ Can't remember.... Please go to Q9

Q8 **Last time you tried to, were you <u>able</u> to get an appointment with a doctor <u>more than</u> 2 full weekdays in advance?**

- ☐ Yes
- ☐ No
- ☐ Can't remember

D. ARRIVING FOR YOUR APPOINTMENT

Q9 **How easy do you find it to get into the building at this GP surgery or health centre?**

- ☐ Very easy
- ☐ Fairly easy
- ☐ Not very easy
- ☐ Not at all easy

Q10 **How clean is this GP surgery or health centre?**

- ☐ Very clean
- ☐ Fairly clean
- ☐ Not very clean
- ☐ Not at all clean
- ☐ Don't know

Q11 **In the reception area, can other patients overhear what you say to the receptionist?**

- ☐ Yes, but I don't mind
- ☐ Yes, and I am not happy about it
- ☐ No, other patients can't overhear
- ☐ Don't know

Q12 **How helpful do you find the receptionists at this GP surgery or health centre?**

- ☐ Very helpful
- ☐ Fairly helpful
- ☐ Not very helpful
- ☐ Not at all helpful

Q13 **How long after your appointment time do you normally wait to be seen?**

- ☐ I don't normally have appointments at a particular time
- ☐ I am normally seen at my appointment time
- ☐ Less than 5 minutes
- ☐ 5 to 15 minutes
- ☐ 16 to 30 minutes
- ☐ More than 30 minutes
- ☐ Can't remember

Q14 **How do you feel about how long you normally have to wait?**

- ☐ I don't normally have to wait too long
- ☐ I have to wait a bit too long
- ☐ I have to wait far too long
- ☐ No opinion/doesn't apply

E. SEEING THE DOCTOR YOU PREFER

Q15 **Is there a particular doctor you prefer to see at this GP surgery or health centre?**

- ☐ Yes..... Please go to Q16
- ☐ No ...... Please go to Q18
- ☐ There is usually only one doctor in my GP surgery or health centre .... Please go to Q18

Q16 **How often do you see the doctor you prefer to see?**

- ☐ Always or almost always
- ☐ A lot of the time Some
- ☐ of the time Never or
- ☐ almost never
- ☐ Not tried at this GP surgery or health centre

Q17 **Was your consultation, <u>which took place on the date referred to in the accompanying letter</u>, with the doctor you prefer to see?**

- ☐ Yes
- ☐ No

F. OPENING HOURS

Q18 **How satisfied are you with the hours that this GP surgery or health centre is open?**

- ☐ Very satisfied
- ☐ Fairly satisfied
- ☐ Neither satisfied nor dissatisfied
- ☐ Fairly dissatisfied
- ☐ Very dissatisfied
- ☐ I'm not sure when my GP surgery or health centre is open

Please turn over ☞

Q19 **As far as you know, is this GP surgery or health centre open…**

Please put an ✕ in one box for each row

	Yes	No	Sometimes	Don't know
... before 8am?	☐	☐	☐	☐
... at lunchtime?	☐	☐	☐	☐
... after 6.30pm?	☐	☐	☐	☐
... on Saturdays?	☐	☐	☐	☐
... on Sundays?	☐	☐	☐	☐

Q20 **Would you like this GP surgery or health centre to open at additional times?**

☐ Yes..... Please go to Q21
☐ No ...... Please go to Section G

Q21 **Which one of the following additional times would you most like this GP surgery or health centre to be open? Please pick one answer showing the time you would most like it to be open.**

☐ Before 8am At
☐ lunchtime After
☐ 6.30pm On a
☐ Saturday
☐ On a Sunday

G. SEEING A DOCTOR AT THE GP SURGERY OR HEALTH CENTRE

Thinking about the consultation, which took place on the date referred to in the letter accompanying this questionnaire...

Q22 **How good was the doctor at each of the following?**

Please put an ✕ in one box for each row

	Very good	Good	Neither good nor poor	Poor	Very poor	Doesn't apply
Giving you enough time	☐	☐	☐	☐	☐	☐
Asking about your symptoms	☐	☐	☐	☐	☐	☐
Listening to you	☐	☐	☐	☐	☐	☐
Explaining tests and treatments	☐	☐	☐	☐	☐	☐
Involving you in decisions about your care	☐	☐	☐	☐	☐	☐
Treating you with care and concern	☐	☐	☐	☐	☐	☐
Taking your problems seriously	☐	☐	☐	☐	☐	☐

Q23 **Did you have confidence and trust in the doctor you saw?**

☐ Yes, definitely
☐ Yes, to some extent
☐ No, not at all
☐ Don't know/can't say

H. YOUR OVERALL SATISFACTION

Q24 **In general, how satisfied are you with the care you get at this GP surgery or health centre?**

- ☐ Very satisfied
- ☐ Fairly satisfied
- ☐ Neither satisfied nor dissatisfied
- ☐ Fairly dissatisfied
- ☐ Very dissatisfied

Q25 **Would you recommend this GP surgery or health centre to someone who has just moved to your local area?**

- ☐ Yes, would definitely recommend
- ☐ Yes, might recommend
- ☐ Not sure
- ☐ No, would probably not recommend
- ☐ No, would definitely not recommend
- ☐ Don’t know

I. SOME QUESTIONS ABOUT YOU

The following questions will help us to see how experiences vary between different groups of the population. We will keep your answers completely confidential.

Q26 **Are you male or female?**

- ☐ Male
- ☐ Female

Q27 **How old are you?**

- ☐ Under 18
- ☐ 18 to 24
- ☐ 25 to 34
- ☐ 35 to 44
- ☐ 45 to 54
- ☐ 55 to 64
- ☐ 65 to 74
- ☐ 75 to 84
- ☐ 85 or over

Q28 **Which of these best describes what you are doing at present?**

If more than one of these applies to you, please ✗ the main ONE only

- ☐ Full-time paid work (30 hours or more each week) .... Please go to Q29
- ☐ Part-time paid work (under 30 hours each week) .... Please go to Q29
- ☐ Full-time education at school, college or university
- ☐ Unemployed
- ☐ Permanently sick or disabled
- ☐ Fully retired from work
- ☐ Looking after the home
- ☐ Doing something else

Please go to Q31

Q29 **In general, how long does your journey take from home to work (door to door)?**

- ☐ Up to 30 minutes
- ☐ 31 minutes to 1 hour
- ☐ More than 1 hour
- ☐ I live on site

Q30 **If you need to see a doctor at your GP surgery or health centre during your typical working hours, can you take time away from your work to do this?**

- ☐ Yes
- ☐ No

Q31 **In general, would you say your health is…?**

- ☐ Excellent
- ☐ Very good
- ☐ Good
- ☐ Fair
- ☐ Poor

Please turn over ☞

Q32 **Do you have any of the following long-standing conditions? Please include problems which are due to old age.**

Please ✗ all the boxes that apply to you

- ☐ Deafness or severe hearing impairment
- ☐ Blindness or severe visual impairment
- ☐ A condition that substantially limits one or more basic physical activities such as walking, climbing stairs, lifting or carrying
- ☐ A learning difficulty
- ☐ A long-standing psychological or emotional condition
- ☐ Other, including any long-standing illness
- ☐ No, I do not have a long-standing condition

Q33 **Are you a deaf person who uses sign language?**

- ☐ Yes
- ☐ No

Q34 **Are you a parent or a legal guardian for any children aged under 16 currently living in your home?**

- ☐ Yes
- ☐ No

Q35 **Do you have carer responsibilities for anyone in your household with a long-standing health problem or disability?**

- ☐ Yes
- ☐ No

Q36 **What is your ethnic group?**

Choose one section from A to E below, then select the appropriate option to indicate your ethnic group

A. White

- ☐ British
- ☐ Irish
- ☐ Any other White background

B. Mixed

- ☐ White and Black Caribbean
- ☐ White and Black African
- ☐ White and Asian
- ☐ Any other Mixed background

C. Asian or Asian British

- ☐ Indian
- ☐ Pakistani
- ☐ Bangladeshi
- ☐ Any other Asian background

D. Black or Black British

- ☐ Caribbean
- ☐ African
- ☐ Any other Black background

E. Chinese or other ethnic group

- ☐ Chinese
- ☐ Any other ethnic group

Q37 **Were you born in the UK?**

- ☐ Yes
- ☐ No

Q38 **What language do you speak most often at home?**

- ☐ English
- ☐ Other (please specify)

THANK YOU FOR YOUR TIME

Please return this questionnaire in the pre-paid envelope provided (no stamp is needed).

If for any reason you do not have a pre-paid envelope, please return the questionnaire using the freepost address below:

GPPS PROGAMME
FREEPOST RRJE-SLSG-RJSY
Peninsula College of Medicine and Dentistry
Primary Care Research Group
Smeall Building, St Lukes Campus
Magdalen Road
Exeter
EX1 2LU

This questionnaire has been developed in conjunction with the Peninsula Medical School and the General Practice and Primary Care Research Unit at the University of Cambridge.

Your practice has asked that we collect any further comments you would like to make about the service they provide.
